# Pharmacokinetic Modeling of Morphine’s Effect on Plasma Concentrations of Ticagrelor and Its Metabolite in Healthy Volunteers

**DOI:** 10.3389/fphys.2021.663170

**Published:** 2021-06-24

**Authors:** Katarzyna Buszko, Krystian Kubica, Eva-Luise Hobl, Piotr Adamski, Kacper Wnuk, Bernd Jilma, Jacek Kubica

**Affiliations:** ^1^Department of Biostatistics and Biomedical Systems Theory, Collegium Medicum in Bydgoszcz, Nicolaus Copernicus University, Toruń, Poland; ^2^Department of Biomedical Engineering, Wrocław University of Science and Technology, Wrocław, Poland; ^3^Department of Clinical Pharmacology, Medical University of Vienna, Vienna, Austria; ^4^Department of Cardiology and Internal Medicine, Collegium Medicum in Bydgoszcz, Nicolaus Copernicus University, Toruń, Poland

**Keywords:** modeling of pharmacokinetics, morphine, ticagrelor, AR-C124910XX, morphine’s effect

## Abstract

This study aimed to build a mathematical model describing the pharmacokinetics of ticagrelor and its active metabolite (AR-C124910XX) in a stable setting with concomitant administration of morphine. The model consists of a set of four differential equations prepared upon the available knowledge regarding the biological processes in the pharmacokinetics of ticagrelor. The set of equations was solved numerically using the Runge–Kutta method. The data were obtained in a double-blind, randomized, placebo-controlled, crossover trial. Twenty-four healthy volunteers received a 180-mg ticagrelor loading dose together with either 5-mg morphine or placebo. Blood samples were analyzed with liquid chromatography–tandem mass spectrometry to assess plasma concentrations of ticagrelor and AR-C124910XX before ticagrelor loading dose and after that 1, 2, 3, 4, and 6 h. The model allowed us to reproduce the experimental results accurately and led us to conclusions consistent with clinical observations that morphine delays the time of maximum drug concentration and that the morphine effect occurs due to decreased gastrointestinal motility. Based on the model, we were able to predict the effect of drug dose on receptor blocking efficacy.

## Introduction

Ischemic heart disease is one of the most important causes of morbidity and mortality worldwide. Acute events cause a significant number of hospitalizations and deaths attributed to coronary artery disease. Modern pharmacotherapy of acute coronary syndrome (ACS) is based on antiplatelet treatment aimed to restrict one of the main pathomechanisms behind this condition, namely excessive activation and aggregation of platelets.

Ticagrelor is a potent, oral antiplatelet agent acting through selective and reversible blockade of platelet P2Y12 receptors, preventing adenosine diphosphate (ADP)-mediated platelet activation and aggregation. According to the latest guidelines of the European Society of Cardiology, ticagrelor is recommended as the first-line treatment for the vast majority of patients presenting with ACS, qualified for both invasive and conservative treatments (Valgimigli et al., 2018). Subsequently, its use in everyday practice increases continuously (Basra et al., 2018).

Morphine is an opioid analgesic that is frequently used in ACS patients to alleviate chest pain, dyspnea, and anxiety symptoms frequently present in this clinical setting. Apart from its beneficial effects, morphine also impedes gastric emptying and intestinal passage, hindering and lagging absorption of orally administered medications (Kubica et al., 2016a). As a result, coadministration of morphine and ticagrelor leads to reduced intestinal intake and impaired antiplatelet effect of this P2Y12 receptor antagonist, which eventually may result in adverse clinical events negatively affecting the ACS prognosis (Aradi et al., 2015; Kubica et al., 2016b). Nevertheless, morphine remains the analgesic of choice in this context with no true alternative, and according to the latest European Society of Cardiology guidelines, it should be considered in ST-elevation myocardial infarction (Ibanez et al., 2018); however, it is also often used in other ACS types.

Evaluation of pharmacokinetic (PK) and pharmacodynamic (PD) profiles of novel drugs in healthy volunteers is a standard procedure during the development and approval processes. This also plays a vital role in the further assessment of drug–drug interactions and their underlying mechanisms. Moreover, it enables verification of strategies designed to overcome detrimental interactions between different substances and/or to improve their PK/PD features.

Mathematical modeling of PK and PD of drugs and drug–drug interactions are performed for over 20 years. The various types of models (compartmental models, non-compartmental models, physiologically based modeling, and semi-mechanistic models) are still being developed. The predictive models are widely used by pharmaceutical companies, especially analyzing *in vitro* data (Rosenbaum, 2017). The development of predictive mathematical modeling of PK and PD of drugs would support personalized medicine.

In mathematical modeling of simulating PK of drugs, the main goal is to formulate mathematical expressions describing alteration of medication concentrations over time. Mathematical models include various parameters that may substantially affect the PK of the drug. Analysis of such parameters in simulations allows evaluation of the influence of different factors on PK. The PK profile of ticagrelor has been widely described in the literature. Evaluation of PK/PD profiles of ticagrelor (regarding single-dose and steady-state PKs) and its metabolite, AR-C124910XX, has been performed in healthy volunteers (Teng and Butler, 2010). Moreover, multiple mathematical models have been proposed to assess PK profile in patients with ACS, prior myocardial infarction, and subjects with liver cirrhosis (Åstrand et al., 2019; Zhang et al., 2019). Apart from evaluating PK profiles in certain groups, mathematical methods have been used to describe the interactions such as between ticagrelor and ritonavir, a potent CYP3A inhibitor, or interaction between ticagrelor and its antidote in mice (Almquist et al., 2016; Marsousi et al., 2016). The PK model presented in this paper is the first mathematical approach to evaluate ticagrelor–morphine interaction in healthy subjects.

## Materials and Methods

### Study Design

Mathematical models depicting PK of ticagrelor and its active metabolite during the first 6 h after a standard 180-mg (i.e., 344.534 μmol) loading dose in healthy subjects with coadministration of intravenous morphine have been created. The model was based on patient-level data originating from a double-blind, randomized, placebo-controlled, crossover trial conducted at the Department of Clinical Pharmacology of the Medical University of Vienna (ClinicalTrials.gov identifier: NCT01369186) (Hobl et al., 2016). The study was approved by the Ethics Committee of the Medical University of Vienna and the Austrian National Competent Authority and was carried out according to Good Clinical Practice Guidelines and the Declaration of Helsinki. Written informed consent was obtained from all participants (*n* = 24). Briefly, healthy volunteers received a 180-mg ticagrelor loading dose together with either 5-mg morphine or placebo (0.9% saline) administered intravenously and subsequently underwent PK evaluation. Next, after a 14-day washout period, participants crossed over to receive a ticagrelor loading dose with placebo if they received morphine with ticagrelor earlier or with morphine if they were allocated to placebo during the first PK assessment. Results of the study, together with a specific description of the methodology, inclusion, and exclusion criteria, were previously published (Hobl et al., 2016).

### Patient Population

The study included 24 healthy adults (10 females and 14 males), aged 27 ± 7 years old, with a body mass index of 23 ± 3 kg/m^2^. Patients on non-steroidal anti-inflammatory drugs, platelet inhibitors or steroids, with known coagulation disorders, relevant impairment of renal or hepatic function, or chronic infectious diseases were not considered for the study. All participants were fasting and abstaining from smoking for at least 4 h before the blood sampling.

### Pharmacokinetic Evaluation

Plasma concentrations of ticagrelor and AR-C124910XX were assessed with liquid chromatography–tandem mass spectrometry. Blood samples were obtained using an intravenous catheter before ticagrelor loading dose and after that every hour until 6 h (0, 60, 120, 180, 240, and 360 min). The values of PK parameters obtained in the study are presented in Table 1 and were published in Hobl et al. (2016).

**TABLE 1:** Pharmacokinetic parameters of ticagrelor and its active metabolite with loading dose equal to 180 mg (Hobl et al., 2016).

	Ticagrelor			Metabolite		
Parameter	Placebo	Morphine	*p*	Placebo	Morphine	*p*
C_max_(ng/mL)	222 (980–1,570)	913 (708–1,137)	0.015	325 (281–399)	242 (280–346)	0.028
T_max_(min)	0 (83–180)	180 (120–240)	0.016	180 (120–240)	240 (180–240)	0.023

### Ticagrelor

Ticagrelor exhibits linear PKs in a dose-dependent manner in healthy volunteers and in a wide spectrum of patients, including those with the chronic coronary syndrome and ACS (Teng, 2012). Ticagrelor is a direct-acting drug that does not require hepatic activation to exert its antiplatelet effect; however, it undergoes metabolism through hepatic CYP3A enzymes (Zhou et al., 2011). AR-C124910XX is the only metabolite of ticagrelor with antiplatelet capability, and its platelet inhibition potential is similar to the parent drug (Teng et al., 2010). After oral intake in stable setting, ticagrelor is promptly absorbed and extensively metabolized into AR-C124910XX, with time to maximal concentration (*t*_max_) 1.3–2 and 1.5–3 h for both compounds, respectively (Teng and Butler, 2010; Teng et al., 2010; Husted et al., 2012). The bioavailability of ticagrelor after oral ingestion is 36%, and AR-C124910XX is present at 35–40% of the parent drug plasma concentration (Husted et al., 2006; Teng et al., 2010; Teng and Maya, 2014). Ticagrelor is excreted mainly with feces and has an elimination half-life of 7–8.5 h, whereas the elimination half-life of AR-C124910XX is 8.5–10 h (Teng and Butler, 2010). Simultaneous administration of morphine and ticagrelor in an acute setting decreases the bioavailability of the latter by one-third, significantly reducing its antiplatelet effect during the first hours after the loading dose (Kubica et al., 2016a; Adamski et al., 2019). These interactions should be considered clinically important, as even up to 50% of patients with acute myocardial infarction treated with ticagrelor receive morphine (Adamski et al., 2017). Similarly, concurrent administration of ticagrelor and morphine in healthy subjects decreases ticagrelor bioavailability by 22%, but with no apparent effect on platelet inhibition (Hobl et al., 2016). Additionally, ticagrelor as well as AR-C124910XX were delayed in healthy volunteers receiving simultaneously ticagrelor loading dose and morphine compared with subjects without additional morphine injection (ticagrelor *t*_max_ : 180 vs. 120 min, *p* = 0.016; AR-C124910XX *t*_max_: 240 vs. 180 min, *p* = 0.023). Moreover, decreased maximal plasma concentration C_max_ of the drug and its active metabolite by 25% with concomitant morphine administration was observed (Hobl et al., 2016). Generally, available data suggest that ticagrelor PK alteration observed with morphine is rather due to decreased and delayed intestinal absorption than metabolic interaction, as these two drugs follow different metabolic pathways (Smith, 2009; Adamski et al., 2018).

### Mathematical Modeling

In our model describing PK of ticagrelor, we have included the following features: the rate of drug release in the intestines, rate of drug penetration from the intestines *via* enterocytes into the circulatory system, dose of the drug, rate of ticagrelor conversion into AR-C124910XX, elimination half-times of the drug and its metabolite, availability of the drug unbound to serum proteins, and, finally, rate of binding of the drug and its active metabolite to P2Y12 receptors of platelets and other cells. The model containing the processes as mentioned earlier can be limited to a two-compartmental model. The first compartment describes changes in the amount of ticagrelor in the intestine. After the intestinal absorption, all processes occur in blood; therefore, it was selected as a second compartment.

**FIGURE 1:**
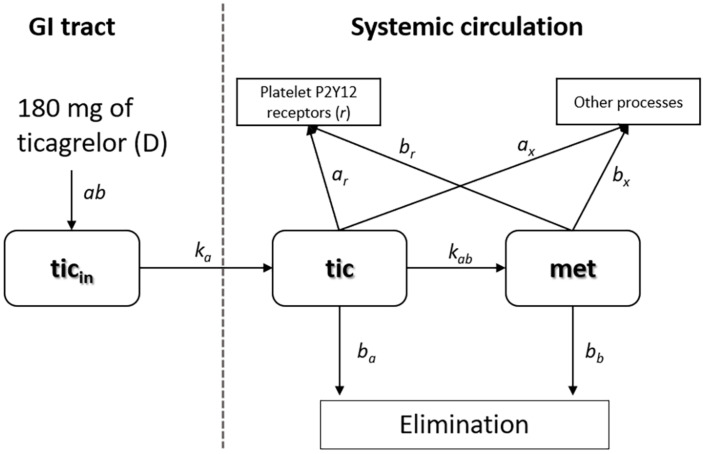
Schematic representation of the two-compartment model of ticagrelor’s pharmacokinetics.

Figure 1 contains a schematic representation of the two-compartment model of ticagrelor’s PKs described in detail in this section. The first compartment reflects intestines, where the absorption of ticagrelor occurs. The second compartment reflects the bloodstream. Other processes include transformation into metabolites (other than AR-C124910XX), binding to non-platelet P2Y12 receptors as well as plasma and tissue proteins. The terms used in the figure are explained later.

Our two-compartmental model consists of four differential equations and has the following form:

(1) $$\frac{{\text{dtic}}_{\text{in}}}{dt}=\left(\text{D}-{\text{tic}}_{\text{in}}\right)\cdot \mathrm{ab}-{\text{k}}_{\text{a}}\cdot {\text{tic}}_{\text{in}}$$

(2) $$\frac{\text{dtic}}{dt}=\frac{{\text{k}}_{\text{a}}\cdot {\text{tic}}_{\text{in}}}{{\text{V}}_{\text{P}}}-{\text{b}}_{\text{a}}\cdot \text{tic}-{\text{k}}_{\text{ab}}\cdot \text{tic}-{\text{k}}_{\text{ar}}\cdot \mathrm{tic}\cdot r-{\text{a}}_{\text{x}}\cdot \text{tic}$$

(3) $$\frac{\text{dmet}}{dt}={\text{k}}_{\text{ab}}\cdot \text{tic}-{\text{b}}_{\text{b}}\cdot \text{met}-{\text{k}}_{\text{br}}\cdot \mathrm{met}\cdot r-{\text{b}}_{\text{x}}\cdot \text{met}$$

(4) $$\frac{\text{dr}}{dt}=-{\text{k}}_{\text{ar}}\cdot \mathrm{tic}\cdot r-{\text{k}}_{\text{br}}\cdot \mathrm{met}\cdot r$$

**FIGURE 2:**
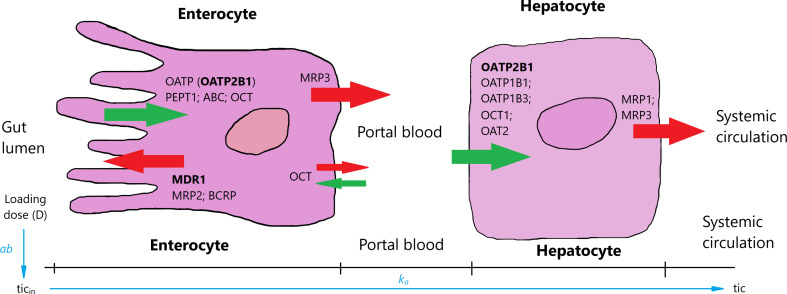
Selected uptake (green arrows) and efflux (red arrows) transporters in enterocytes and hepatocytes [based on Giacomini et al. (2010) and Drozdzik et al. (2014)]. Ticagrelor is absorbed in the intestines and through portal blood gets to hepatocytes and further to systemic circulation. Transporters inhibited by ticagrelor are in bold. OATP, organic anion transporting polypeptide; PEPT1, peptide transporter 1; ABC, ATP-binding cassette transporter; OCT, organic cation transporter; MDR1, multi-drug resistance 1; MRP1–3, multidrug resistance-associated protein 1–3; BCRP, breast cancer resistance protein; OAT2, organic anion transporter 2.

The first equation describes the rate of changes in ticagrelor’s quantity in the intestine expressed in micromole per minute, whereas Equations 2–4 represent the rate of changes in ticagrelor, AR-C124910XX, and free P2Y12 receptor concentrations expressed in micromole per milliliter per minute. As the drug is administered in the form of a tablet, the entire dose (D) initially will be in the stomach and subsequently will be gradually displaced into the intestine. We assumed that the speed of this process slows down as the amount of drug in the stomach decreases, and the process can be described in the form: (*D* − *tic*_in_) × *ab*, where *D* is the dose, tic_in_ the current amount of ticagrelor in the intestine, and *ab* is the kinetic constant. This process is accompanied by a parallel movement of the drug to the systemic circulation. Ticagrelor is a substrate and weak inhibitor of P-glycoprotein (Teng and Butler, 2013); furthermore, its inhibitory properties regarding organic anion transporting polypeptide 2B1, widely expressed in many tissues, including the entire human intestine, has been suggested (Khuri et al., 2017). These and other selected transporters expressed in enterocytes and hepatocytes are shown in Figure 2. Proteins involved in the transport of ticagrelor through enterocyte membrane remain unknown, and although the influence of liver-specific OATP1B1 transporter on ticagrelor’s PKs may suggest a possible role of OATP-mediated transfer from the gut lumen to the blood, it may play only a minor role because, as we mentioned before, ticagrelor exhibits linear PKs (Varenhorst et al., 2015). As a result, the rate of transportation from the intestine to the systemic circulation is expressed as a first-order with effective constant k_a_ [min^–1^] (Figure 2). In summary, the rate of ticagrelor change in the intestine can be written as (*D-tic*_in_) × *ab* − *k*_a_ × *tic*_in_, what is equivalent to: *D* × *ab* − (*ab*+ *k*_a_) × *tic*_in_—which means that the ticagrelor income to the intestine is a process of zero-order with the constant *D* × *ab*. As a result, *D* × *ab* × *t*_in_ corresponds to the amount of the drug absorbed to the systemic circulation, which should be equal to D × F (where F is bioavailability of ticagrelor).

In the second compartment, we considered changes in concentrations of ticagrelor, its active metabolite (AR-C124910XX), and P2Y12 receptors binding the drug and its metabolite (Equations 2–4). To consider the rate of concentration change in plasma, a quantity of ticagrelor transported from the intestines at a constant rate *k*_a_, which should be divided by a volume of plasma *V*_p_ (in Equation 2 the first term). Based on previous reports and available data, we assumed that the value of *V*_p_ equals 2,700 cm^3^, i.e., 60% of blood volume. While describing changes in ticagrelor plasma concentration (Equation 2) apart from its transfer from the enterocyte *via* the portal blood, we should also take into account: elimination rate, rate of its transformation into the active metabolite, binding to platelet and extra platelet P2Y12 receptors, and plasma and tissue fluid proteins (Figure 1). The second term in Equation 2 :−b_a_⋅tic describes the rate of ticagrelor elimination with the half-time τ_a_ = 7 h (i.e., 420 min) (Teng and Butler, 2010). For the first-order reaction, the elimination rate constant b_a_ is bound with the half-time by expression: b_a_ = ln(2)/τ_a_. The third term: −k_ab_⋅tic is responsible for the kinetics of ticagrelor transformation into its active metabolite, where k_ab_ is the effective constant rate, as this reaction is controlled by two enzymes: CYP3A4 and CYP3A5. The rate of ticagrelor binding by the platelet P2Y12 receptors is described by the expression: −k_ar_⋅*tic*⋅r, where k_ar_ is the kinetic rate constant, r is concentration of platelet P2Y12 receptors. The last expression −a_x_⋅tic in Equation 2 describes the binding of the drug by extra platelet P2Y12 receptors as well as plasma and tissue fluid proteins with the kinetic constant a_x_. Due to the large number of proteins with which ticagrelor can bind, we did not consider reducing the pool of these proteins in this process.

The active metabolite of ticagrelor has an antiplatelet effect similar to the parent drug; therefore, its PK has to be included in the presented model. The equation describing the rate of concentration changes of AR-C124910XX (Equation 3) includes: conversion rate of ticagrelor into its active metabolite (k_ab_⋅tic), elimination rate of the metabolite with the half-time τ_b_ = 8.5 h (i.e., 510 min) (Teng and Butler, 2010) with elimination rate constant b_b_equal to 1.35 × 10^–3^ min^–1^, rate of metabolite binding to platelet P2Y12 receptors (−k_br_⋅*met*⋅r), P2Y12 receptors of other cells and proteins, with the kinetic constant b_x_ (Equation 3). Similar to the process of ticagrelor binding to the receptors of other cells and proteins, due to their large amount, we did not take into account the reduction of their amount.

We assumed that the rate of free P2Y12 receptor concentration change depends on the current concentration of both ticagrelor and its metabolite. These relationships are represented by Equation 4, where −k_ar_⋅*tic*⋅r is the rate of binding ticagrelor by platelet P2Y12 receptors r with the kinetic constant K_ar_ and the rate of binding metabolite (−k_br_⋅*met*⋅r) with kinetic constant k_br_.

Equations 2–4 express the rate of concentration changes; therefore, consistently, the number of receptors should also be expressed in micromole per milliliter. One mole of a substance contains the Avogadro number of elements, i.e., 6.021 × 10^23^, and the number of platelets within the normal range is (140–400) × 10^6^ platelets/ml, which corresponds to (2.33–6.66) × 10^–10^ [μmol/ml]. We assumed that the average number of P2Y12 receptors per statistical platelet is 425 (Ohlmann et al., 2013); thus, the concentration of platelet P2Y12 receptors in a healthy subject should be (0.99–2.83) × 10^–7^ [μmol/ml].

To solve Equations 1–4 numerically, we have applied the Runge–Kutta method, coded in MATLAB (version R2017a, solver ode45).

In the first step, we examined the impact of changing individual parameters on changes in *tic*_in_, *tic*, *met*, and *r*, which allowed us to find the range of variability of individual parameters leading to an approximate agreement with the experimental results. Because, in our model, the number of parameters exceeds the number of degrees of freedom of the system under study, it should be expected that there are many sets of parameter values leading to the fit to the experimental results. However, by imposing certain knowledge-based limitations, a choice can be restricted. As the experimental studies were performed on the same group of healthy volunteers, it can be assumed that the elimination rate constants of ticagrelor (*b*_a_) and its metabolite (*b*_b_) will be represented by the mean values and will not be changed. For the same reason, while searching for the optimal set of parameters, we chose a solution for which the rate of conversion of ticagrelor into an active metabolite (*k*_ab_ constant), as well as the binding constants with the platelet receptors (*k*_ar_ and *k*_br_) and with other receptors and proteins (*a*_x_ and *b*_x_) would not differ significantly for both experiments (placebo and morphine). The *k*_ar_ and *k*_br_ parameters take such values that the decrease in the number of free platelet receptors after 1 h after drug administration was similar to the decrease in platelet reactivity in the ADP-induced test shown in Kubica et al. (2016b). In line with these assumptions and available knowledge, morphine does not compete with ticagrelor or its metabolite for binding sites, nor does it affect the rate of conversion of ticagrelor into a metabolite. In accordance with the adopted limitations, in the next step, we searched for a set of optimal parameter values leading to an optimal match to the experimental results by analyzing all the solutions for all combinations of parameters in specified intervals with a specified step. As a step, we assumed a unit change of all parameters within the adopted limits. We assumed that the best-fitted curve represents the minimum value of the sum of squares of deviations (SSD) between theoretical and experimental data [min(SSD)].

## Results

**FIGURE 3:**
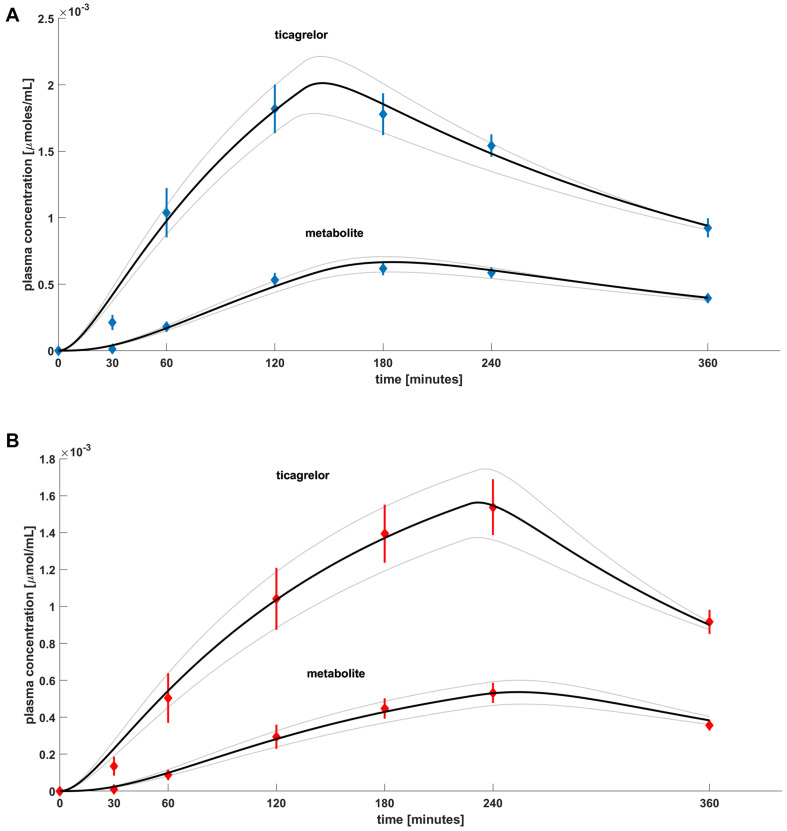
Plasma concentration for **(A)** healthy volunteers after 180-mg ticagrelor loading dose and **(B)** healthy volunteers after 180-mg ticagrelor loading dose administered with 5-mg morphine, mean and SEM of experimental data. Diamonds represent mean of experimental data. Black lines are best-fitted curves from model, and gray lines represent family of possible solutions.

The main results of our investigations performed on the set of equations (Equations 1–4) are presented in Figures 3A,B. The figure shows plasma concentrations of ticagrelor and its metabolite (AR-C124910XX) in placebo (Figure 3A) and morphine (Figure 3B) groups in measurement time points together with the results obtained from the model. Ticagrelor and its metabolite plasma concentrations measured in healthy volunteers after a 180-mg ticagrelor loading dose are plotted as blue diamonds with lines, which represent the mean and standard error of the mean (SEM) of the data (Figure 3A). The plasma concentrations measured in healthy volunteers after a 180-mg ticagrelor loading dose given with 5 mg morphine are plotted as red diamonds with lines and also represent mean and SEM (Figure 3B). To find the optimal fit for the results of clinical trials, we estimated the intervals of possible values of the parameters included in the model (Equations 1–4). For the considered dose (*D*), which was 180 mg, i.e., 344.4526 μmol of ticagrelor, we studied the solutions for both groups’ placebo (P) and morphine (M) by changing the parameters in the intervals and with the steps listed in Tables 2A,B. The numerical calculations performed for both studied groups lead us to three solutions representing the optimal fit to the mean values of the experimental points and those values minus plus SEM (Figure 3). The search for optimal values of these parameters was conducted simultaneously for ticagrelor and AR-C124910XX. For all fitted curves, the SSD took values of the order of 10^–8^ [μmol/cm^3^]^2^.

**TABLE 2:** Estimated intervals of possible values of parameters of model (Equations 1–4) for **(A)** healthy volunteers after 180-mg ticagrelor loading dose (344.453 μmol) and **(B)** healthy volunteers after 180-mg ticagrelor loading dose administered with 5-mg morphine.

		Experimental mean values – SEM		Experimental mean values		Experimental mean values + SEM	
Parameter	Step	Interval	Optimal value	Interval	Optimal value	Interval	Optimal Value
**(A)**							

*t*_in_ [min]	1	130–140	129	130–140	136	130–140	136
*k*_a_ [min^–1^]	1 × 10^–4^	(30–40) × 10^–4^	33 × 10^–4^	(30–40) × 10^–4^	38 × 10^–4^	(40–50) × 10^–4^	43 × 10^–4^
*k*_ab_[min^–1^]	1 × 10^–3^	(8–14) × 10^–3^	10 × 10^–3^	(8–14) × 10^–3^	10 × 10^–3^	(8–14) × 10^–3^	10 × 10^–3^
*a*_x_ [min^–1^]	1 × 10^–3^	(60–70) × 10^–3^	64 × 10^–3^	(60–70) × 10^–4^	66 × 10^–3^	(60–70) × 10^–3^	66 × 10^–3^
*b*_x_ [min^–1^]	1 × 10^–3^	(20–30) × 10^–3^	26 × 10^–3^	(20–30) × 10^–3^	26 × 10^–3^	(20–30) × 10^–3^	27 × 10^–3^
*ab* [min^–1^]	1 × 10^–4^	(38–42) × 10^–4^	40 × 10^–4^	(38–42) × 10^–4^	40 × 10^–4^	(38–42) × 10^–4^	40 × 10^–4^

**(B)**							

*t*_in_ [min]	1	220–230	225	220–230	227	225–235	232
*k*_a_ [min^–1^]	1 × 10^–4^	(35–45) × 10^–4^	39 × 10^–4^	(40–50) × 10^–4^	48 × 10^–4^	(50–60) × 10^–4^	58 × 10^–4^
*k*_ab_[min^–1^]	1 × 10^–3^	(8–14) × 10^–3^	11 × 10^–3^	(8–14) × 10^–3^	11 × 10^–3^	(8–14) × 10^–3^	11 × 10^–3^
*a*_x_ [min^–1^]	1 × 10^–3^	(60–70) × 10^–3^	65 × 10^–3^	(60–70) × 10^–3^	65 × 10^–3^	(60–70) × 10^–3^	65 × 10^–3^
*b*_x_ [min^–1^]	1 × 10^–3^	(25–35) × 10^–3^	29 × 10^–3^	(25–35) × 10^–3^	29 × 10^–3^	(25–35) × 10^–3^	29 × 10^–3^
*ab* [min^–1^]	1 × 10^–4^	(16.6–17.5) × 10^–4^	17.2 × 10^–4^	(16.6–17.5) × 10^–4^	17.1 × 10^–4^	(16.6–17.5) × 10^–4^	17.0 × 10^–4^

Taking into account the personal variability of the subjects, the parameters for optimal matches showed variability: *t*_in_ (7 min), *k*_a_ (10^–3^ min^–1^), *k*_ab_ (0), *a*_x_ (2 × 10^–3^ min^–1^), *b*_x_ (1 × 10^–3^ min^–1^), and *ab* (0) for group P. Analogous variations of parameters for the M group are as follows: *t*_in_ (7 min), *k*_a_ (1.9 × 10^–3^ min^–1^), *k*_ab_ (0), *a*_x_ (0 min^–1^), *b*_x_ (0 min^–1^), and *ab* (0.2 × 10^–4^ min^–1^).

To obtain the same effect of inhibiting platelets as observed in ADP-induced platelet aggregation studies (Kubica et al., 2016b), parameters *k*_ar_ and *k*_br_ were set to 40 cm^3^ μmol^–1^ min^–1^.

We applied the presented model containing optimal values of parameters to assess the effect of changing drug dose on receptor blocking efficacy. In the simulations, we used the following doses of ticagrelor: 360, 180, 135, 90, and 45 mg. The analysis allowed us to predict responses to receptor inhibition, expressed as a percentage of blocked platelet receptors, 1 and 2 h after administration of ticagrelor in both the P and M groups. These results are presented in Table 3.

**TABLE 3:** Estimated percentage of blocked platelet receptors depending on dose based on mathematical model (Equations 1–4) 1 and 2 h after drug administration.

Dose (mg)	% of blocked platelet receptors (group P)		% of blocked platelet receptors (group M)	
	1 h after administration	2 h after administration	1 h after administration	2 h after administration
360	90.5	99.9	73	99.7
180	69.3	99.5	48.1	95.2
135	58.7	98.2	38.8	89.8
90	44.5	93.2	27.5	76.2
45	25.5	74	15.1	53.3

## Discussion

The presented mathematical model of ticagrelor’s PKs in healthy volunteers after the standard loading dose given with or without morphine reflects the experimental data accurately. The current model reflects previously published considerations and conclusions regarding the time course of ticagrelor and its metabolite plasma concentrations in healthy volunteers (Hobl et al., 2016; Kubica et al., 2016b). These papers mainly focused on the negative impact of morphine on the PKs of ticagrelor when these two drugs are administered concomitantly. In our model, we have also observed the “morphine’s effect” responsible for the shift of ticagrelor and its metabolite concentration curves (Figures 3A,B). We attempted to account for it by analyzing changes in parameters that correspond to biological processes included in the model. When we compared values of parameters leading to the optimal fit of theoretical curves and experimental data for healthy volunteers taking the loading dose of 180-mg ticagrelor and the same group receiving ticagrelor and morphine simultaneously (Figures 3A,B), we noticed that parameters *t*_in_ and *k*_a_ have higher values and *ab* has smaller values for the group that received ticagrelor together with morphine. These parameters are responsible for the rate of transportation from the gut lumen to the blood (k_a_), the time (t_in_), and the rate (*ab*) of the drug transport from gastric juice to the intestines; therefore, we have concluded that the delay in PKs of ticagrelor caused by morphine most likely results from decreased gastrointestinal motility, which is a known adverse effect of morphine. These changes lead to an increase in the gradient of ticagrelor concentration at the border of the intestinal lumen and the enterocyte membrane. Due to the chemical structure of the ticagrelor molecule, it is unlikely that the drug crosses the enterocyte membranes by simple diffusion. Carrier-mediated transport is more likely, where concentration gradient acts as a thermodynamic stimulus. Thus, according to our simulations, an increase in the value of the parameter t_in_ is responsible for the delay of intestine peristalsis, which leads to an increase in the gradient of ticagrelor and is expressed by a slight increase of k_a_. Comparisons of parameters for optimal matches also indicate a significant decrease in the *ab* value responsible for the transportation of the drug to the intestine, which may be due to the morphine’s effect on gastric emptying. Based on our results, the absorbed amount of ticagrelor equals to D × ab × *t*_in_ = 177.6 μmol in group P and 133.3 μmol in group M compared with loading dose = 344.45 μmol. These values correspond with the bioavailability of ticagrelor (36%, 95% confidence interval: 30–42%), and differences may be explained by interindividual variability, potential excretion into intestines *via* P-gP-mediated efflux (as ticagrelor is a substrate of this transporter), or direct transport from the liver into the bile (Teng and Maya, 2014). The higher value observed in the placebo group indicates that coadministration of morphine reduces the drug exposure by approximately 20% (Hobl et al., 2016). Importantly, ticagrelor also interacts with extra-platelet P2Y12 receptors located, for instance, in the nervous system, vascular smooth muscle cells, leukocytes, macrophages, and microglial and dendritic cells (Ohlmann et al., 2013). However, similar values of parameters including this interaction (*a*_x_ and *b*_x_) in groups P and M suggest that morphine does not affect ticagrelor’s binding to those receptors.

The presented model was built based on available knowledge regarding the biological processes in PKs of ticagrelor. Based on the created model, we were able to reproduce the experimental results accurately. Moreover, the model allowed us to predict the platelet receptor inhibition efficacy depending on the dose of the drug. Prediction of response to ticagrelor based on doses in the range from 45 to 360 mg, presented in Table 3, indicates that the percentage of inhibited P2Y12 receptors correlates with the dose of the drug similarly as shown in a study by Teng and Butler (2010). Moreover, the addition of intravenous morphine visibly affects this process, especially shortly after the administration of ticagrelor. Consistent with a study (Parodi et al., 2014) that included ST-elevation myocardial infarction patients, doubling ticagrelor’s loading dose to 360 mg in healthy subjects does not significantly affect receptor inhibition compared with the standard dose in the placebo group. However, in the morphine group, the predicted inhibition of P2Y12 receptors 1 h after the 360-mg dose is similar to the one obtained in the placebo group after 180 mg of ticagrelor, which suggests that increased dose of ticagrelor may improve its PDs in patients who received morphine. Further studies are warranted to verify these findings in clinical settings.

### Limitations

In the presented model, we described the process of ticagrelor transport from the gastrointestinal tract to the circulation as simple diffusion. However, the ticagrelor molecule is relatively large and most likely requires carrier-mediated transport across membranes of enterocytes. The transport rate would depend linearly on the concentration of the transported substance until all carriers are involved. If the amount of transported substance exceeds the number of active carriers, then the speed of this process depends only on the number of transporter molecules. Because the number of transported molecules decreases over time, the transport speed again depends on the amount of transported molecules. However, incorporating this process into the model would require assessing the number of carrier molecules that are not stable over time. Additionally, as binding to tissue fluid and plasma proteins has been expressed with parameters *a*_x_ and *b*_x_, we decided to refer to plasma volume instead of volume of distribution while describing PKs of ticagrelor and its metabolite.

## Data Availability Statement

The raw data supporting the conclusions of this article will be made available by the authors, without undue reservation.

## Ethics Statement

The studies involving human participants were reviewed and approved by Ethics Committee of the Medical University of Vienna and the Austrian National Competent Authority and registered at ClinicalTrials.gov (NCT01369186). Written informed consent to participate in this study was provided by the participants’ legal guardian/next of kin.

## Author Contributions

KK created the model, performed numerical calculations, and wrote the manuscript. KB analyzed the data, discussed the model, prepared the figures, and wrote the manuscript. PA discussed the model and wrote the manuscript. KW discussed the model and prepared the figure. E-LH and BJ conceived and designed the experiments. JK supervised the work and conceived and designed the experiments. All authors have revised the manuscript critically and approved the final version of the manuscript.

## Conflict of Interest

The authors declare that the research was conducted in the absence of any commercial or financial relationships that could be construed as a potential conflict of interest.
